# Myxopapillary Ependymoma of the Sacrum

**DOI:** 10.5334/jbsr.3129

**Published:** 2023-05-04

**Authors:** Arnaud R. Goossens, Annelies Van Beeck, Filip M. Vanhoenacker

**Affiliations:** 1 Resident at AZ Sint Maarten Mechelen and Ghent University Hospital, Belgium; 2 Department of Orthopedic Surgery, Antwerp University Hospital, Belgium; 3 Faculty of Medicine and Health Sciences, University of Antwerp, Belgium; 4 Department of Radiology, AZ Sint-Maarten Mechelen and Antwerp University Hospital, Belgium; 5 Faculty of Medicine and Health Sciences, University of Antwerp and Ghent University, Mechelen, Belgium

**Keywords:** myxopapillary ependymoma, sacrum, spine, MRI, CT

## Abstract

**Teaching Point:** Myxopapillary ependymoma presenting as a highly destructive lesion in the sacrum is rare but should be included in the differential diagnosis.

## Case History

A ‘16-year-old’ versus ‘>50 years old’ female is referred for a magnetic resonance imaging (MRI) examination of the lumbosacral spine because of progressive dullness and pain in the tailbone.

**Figure 1:**
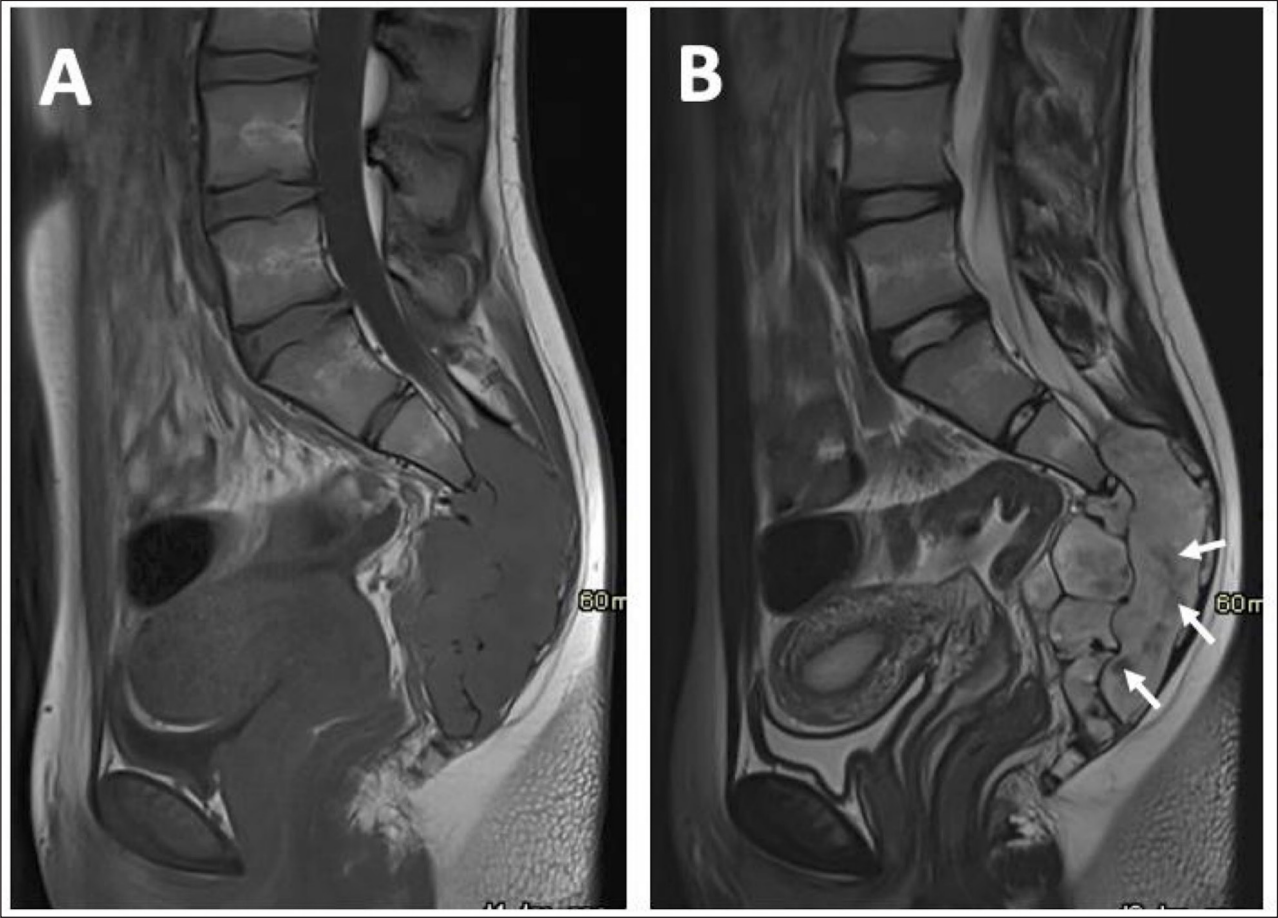
**(A)** Midsagittal T1-WI; **(B)** Midsagittal T2-WI.

**Figure 2:**
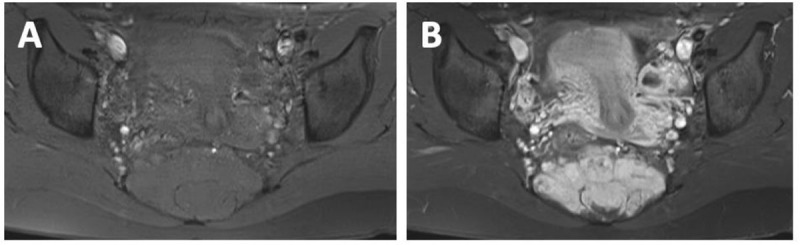
**(A)** Axial T1-WI with spectral fat suppression; **(B)** Axial T1-WI with spectral fat suppression and gadolinium-contrast enhancement.

**Figure 3:**
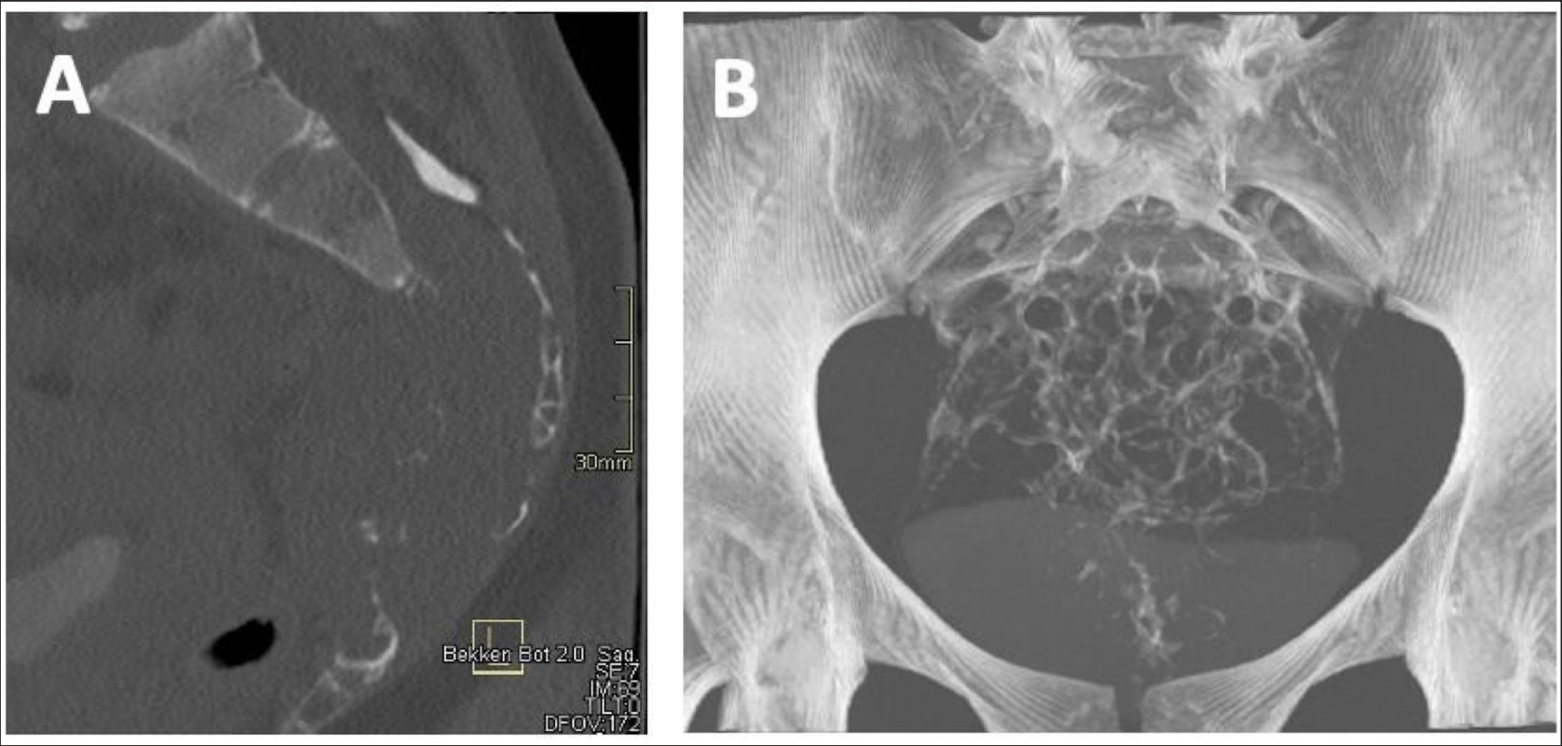
**(A)** Midsagittal bone-window CT; **(B)** Coronal MIP CT.

MRI reveals a multilocular, well-circumscribed lesion arising from the sacral spinal canal at the level S3 to S5 extending into the presacral space through the neuroforamina. The mass is isointense to the spinal cord on T1-weighted images (WI) (Figure 1A) and slightly hyperintense with central hypointense areas (Figure 1B, arrows) on T2-WI. There is predominantly homogeneous enhancement after the administration of gadolinium-based contrast (Figure 2A–B). Extensive cortical destruction is better appreciated on computed tomography (CT) (Figure 3A–B). A CT-guided biopsy is performed and immunohistological diagnosis of myxopapillary ependymoma is made, followed by peacemeal surgery for nerve sparing.

## Comments

Myxopapillary ependymoma (MPE) is a mucoid tumor that typically arises from the lower spinal cord or filum terminale. Location in the sacrum is rare. It tends to be more commonly seen in male patients with a mean age of 36.

Small MPE are difficult to detect on CT because they are isodense to the spinal cord. Larger lesions may cause pressure erosion on the spinal canal or neuroforamina. Extensive cortical destruction and soft tissue extension has specifically been reported in sacral lesions [1].

On MRI, MPE is usually T1-isointense and T2-hyperintense relative to the spinal cord, with vivid homogeneous enhancement after gadolinium-contrast administration. MRI is the best diagnostic imaging tool to evaluate local tumor extension.

The most important parameters for the differential diagnosis of large destructive sacral lesions are age and MR-imaging features [1]. Destructive sacral lesions are mostly seen in late adulthood (‘16-year-old’ versus ‘>50 years old’) and in elderly, especially chordoma. Multiple myeloma and metastasis are also more frequent in this age range. For young and middle-aged adults, osseous giant cell tumor (GCT) and chondrosarcoma are more common. Aneurysmal bone cyst (ABC), Ewing sarcoma and secondary bone lymphoma are typically seen in children and adolescents.

Nearly all destructive sacral lesions are hypo- to isointense on T1-WI and iso- to hyperintense on T2-WI relative to the spinal cord with enhancement of solid components. Fluid/fluid levels can be seen in ABC, osseous GCT and metastasis. Blooming artefact on T2* sequences due to hemosiderin deposition is frequently seen in osseous GCT. Calcifications, More easily identified on CT, can be present in chordoma and osteosarcoma.

Biopsy and histopathologic examination are required for final diagnosis.

The treatment of choice is gross total resection, with or without adjuvant radiation therapy.

## References

[1] Cihangiroglu M, Hartker FW, Lee M, Sehgal V, Ramsey RG. Intraosseous sacral myxopapillary ependymoma and the differential diagnosis of sacral tumors. Journal of Neuroimaging. 2001 Jul; 11(3): 330–2. DOI: 10.1111/j.1552-6569.2001.tb00058.x 11462306

